# Long-Term Safety and Performance of a National Pericardium Organic Valvular Bioprosthesis in the Brazilian Public Health System: Retrospective Analysis Up To 26 Years of Follow-up

**DOI:** 10.21470/1678-9741-2024-0405

**Published:** 2025-08-22

**Authors:** Roberto Vito Ardito, Renata Andrea Barberio Bogdan

**Affiliations:** 1 Department of Cardiac Surgery, Instituto de Moléstias Cardiovasculares, São José do Rio Preto, São Paulo, Brazil

**Keywords:** Heart Valve Prosthesis, Aortic Valve, Mitral Valve, Public Health, Retrospective Study.

## Abstract

**Objective:**

To evaluate the long-term performance of a bovine pericardium valve prosthesis in individuals who required valve replacement in the Brazilian public health system.

**Methods:**

Medical records of patients having mitral or aortic valve replacement with bovine pericardium valve prostheses between 1978 and 1994 at a Brazilian hospital were reviewed in this retrospective study. Safety was assessed through the complications and serious adverse events rates in the early and long terms. Successful valve replacement was defined by absence of complications and serious adverse events up to 30 days after surgery.

**Results:**

A total of 439 surgeries were performed in 382 patients with a mean age of 46.45 ± 13.93 years. Mean follow-up time was 6.26 years (up to 26.13 years). Rheumatic etiology was present in 83.5% of the cases. Mitral valve replacement was the most performed surgery. Five complications in five patients were recorded up to 30 days after surgery, and the rate of serious adverse events for the same period was 10.3%. Successful valve replacement rate was 90.7%. Postoperative complications were reported during the follow-up period in 29.6% of the procedures, being calcification the most common with 17.3%.

**Conclusions:**

Despite the young age of the patients, safety outcomes were in accordance with what is reported in the literature for bioprostheses, with acceptable complication, serious adverse events, and freedom from reintervention rates.

## INTRODUCTION

**Table:** 

Abbreviations, Acronyms & Symbols	
A	= Aortic
AVR	= Aortic valve replacement
BMI	= Body mass index
BVP	= Bovine Pericardium Organic Valvular Bioprosthesis
M	= Mitral
MVR	= Mitral valve replacement
NYHA	= New York Heart Association
SAEs	= Serious adverse events
SD	= Standard deviation

Heart valve diseases account for nearly one-third of all heart surgeries performed worldwide^[1]^, this high prevalence is related to the increasing elderly population globally. Valve replacement is the most common valvular surgery in the United States of America^[2]^ and the second most common in the United Kingdom^[3]^.

In Brazil, valve prosthesis implantation ranks as the second most frequent procedure in highly complex cardiovascular surgeries according to data of the Brazilian public health system^[4]^. That can be associated with the high incidence of rheumatic heart disease in the country, which even these days reaches almost 30.000 cases every year. As a result, it has been reported that 70% of patients with acute rheumatic fever develop carditis, and one-third of cardiovascular surgeries performed in Brazil are attributable to sequelae of rheumatic heart disease^[5]^.

Valve replacement becomes necessary when one or more natural heart valves fail to function properly. It is considered the gold standard surgery for aortic valve diseases^[6]^ and has been improving the survival and quality of life of the patients for the last six decades^[7]^.

There are two main types of heart valve replacement: biological valves, made from animal tissue such as bovine pericardium, and mechanical valves, composed of synthetic materials^[8]^.

The Bovine Pericardium Organic Valvular Bioprosthesis (BVP) was first used clinically in 1977 and has since demonstrated excellent hemodynamic performance, durability, and a low incidence of valve-related adverse events^[8]^.

Therefore, the aim of the present study was to retrospectively analyze clinical data on the long-term performance of Braile BVP in individuals who required replacement of their native or prosthetic valves (aortic or mitral).

## METHODS

### Study Design and Sample

The present retrospective study was approved by the Ethics Committee under the approval number 3.920.059 and conducted in accordance with the principles of the Helsinki Declaration, ISO 14155:2011, and applicable Brazilian laws and recommendations.

Medical records of patients having mitral or aortic valve replacement with Braile BVP between 1978 and 1994 at Hospital do Coração of the Instituto de Moléstia Cardiovasculares (São José do Rio Preto, Brazil) were reviewed. Patients younger than 18 years old were excluded from this analysis.

### Prosthesis Information

Braile BVP is made from bovine pericardium, treated with glutaraldehyde, and preserved with 4% formaldehyde, ensuring it has the necessary characteristics of resistance, flexibility, and non-antigenicity. The bioprosthesis is constructed on a polyacetal stent, covered with bovine pericardium, to which the cusps are affixed. A special stainless-steel wire, inserted externally into the support ring, reinforces the base of the ring and allows the prosthesis to be identified via radiological examination. The prosthesis is indicated for the replacement of the mitral, aortic, tricuspid, or pulmonary valves and is available in diameters of 19, 21, 23, 25, 27, 29, 31, and 33 mm^[9]^.

### Study Endpoints

Safety was assessed through the complications and serious adverse events (SAEs) rates up to 30 days after surgery. Successful valve replacement was defined by absence of complications and SAEs up to 30 days after surgery.

Efficacy was assessed through the evaluation of patients’ New York Heart Association (NYHA) classification before and after surgery.

### Statistical Analysis

Descriptive analyses were performed for all clinical findings that were recorded in the medical files: demographic data, medical history, and intra and postoperative information. Categorical data are summarized as absolute frequencies (n) and percentages (%), whereas continuous data are presented as mean (± standard deviation), or median and range.

Early-term and long-term complication rates, as well as the incidence of SAEs, were determined by analyzing the number of reported events occurring within 30 days after surgery and in the subsequent period, relative to the total number of surgeries performed for each type of replaced valve.

To assess cumulative survival, freedom from SAEs during the first year, and the freedom from reintervention during the follow-up period, Kaplan-Meier was used. The chi-square log-rank test was applied to compare curves between groups. All analyses were performed using the IBM Corp. Released 2017, IBM SPSS Statistics for Windows, version 25.0, Armonk, NY: IBM Corp. software.

## RESULTS

A total of 439 surgeries were performed in 382 patients with a mean age of 46.45 ± 13.93 years, ranging between 18 and 81 years. Patients younger than 70 years old accounted for 96.8% of the sample. Male patients comprised 50.2% of the sample while female patients represented 49.8%. The mean body mass index was 22.7 ± 4.27 kg/m2. Mean follow-up time was 6.26 years (up to 26.13 years).

When patients’ medical history was evaluated, the majority were non-smokers (80.6%) and did not have coronary disease (95.5%), arterial hypertension (63.3%), fibrillation (71.3%), cardiac arrhythmia (62.2%), renal dysfunction/insufficiency (99.2%), diabetes (97.3%), or history of stroke (94.7%). However, regarding pulmonary hypertension, it was present in most patients (53.5%). The main etiology of valve disease was rheumatic fever, which was present in 83.5% of the patients. Demographic characteristics of the evaluated patients are presented in Table 1.

**Table 1:** Demographic characteristics. Analysis per number of patients (n = 382).

Variable	
Age (years), mean ± SD	46.45 ± 13.93
BMI (kg/m^2^), mean ± SD	22.70 ± 4.27
Mean follow-up period (years) (min - max)	6.26 (0 - 26.13)
Sex (n = 382), n (%)	
Male	192 (50.2)
Female	190 (49.8)
Age group, n (%)	
≥ 70 years	12 (3.2)
< 70 years	370 (96.8)
Etiology (n = 248), n (%)	
Rheumatic	207 (83.5)
Myxomatous	41 (16.5)
Smoking habits (n = 382), n (%)	
Yes	74 (19.3)
Coronary disease (n = 382), n (%)	
Yes	16 (4.1)
Pulmonary hypertension (n = 380), n (%)	
Yes	203 (46.6)
Arterial hypertension (n = 379), n (%)	
Yes	139 (36.7)
Previous stroke (n = 380), n (%)	
Yes	20 (5.3)
Fibrillation (n = 380), n (%)	
Yes	109 (28.7)
Cardiac arrhythmia (n = 381), n (%)	
Yes	144 (37.8)
Renal dysfunction/insufficiency (n = 380), n (%)	
Yes	3 (0.8)
Diabetes (n=379), n (%)	
Yes	10 (2.6)

BMI=body mass index; SD=standard deviation

Data related to the surgeries are presented in Table 2. Of the total surgeries performed, 76.8% were first valve replacement procedures, 20% were second surgeries, 3% were third surgeries, and only 0.2% were fourth surgeries. Aortic valve replacement accounted for 35.7% of the procedures, while mitral valve replacement represented 55.4%. Surgeries involving both mitral and aortic valve replacement comprised 8.9% of the total. The most commonly used prosthesis size for aortic valve replacement was 23 mm, followed by 25 mm. For mitral valve replacement, the most used prosthesis size was 29 mm, followed by 27 mm. One hundred and four (23.7%) surgeries were performed with an associated procedure, being tricuspid valve plasty the most frequently performed associated surgery, occurring in 58.6% of cases. Postoperative complications were reported during the follow-up period in 29.6% of the procedures, with calcification being the most common at 17.3%, followed by endocarditis (4.1%) and rupture (3.2%). Throughout the entire follow-up period, 176 SAEs (40%) were reported, and new surgery was deemed necessary in 11.1% of the cases.

**Table 2:** Intra and postoperative data. Analysis per number of surgeries (n=439).

Variable	n (%)
Previous valve replacement surgery (n = 439)	
No	337 (76.8)
1 surgery	88 (20.0)
2 surgeries	13 (3.0)
3 surgeries	1 (0.2)
Valve (n = 439)	
Aortic	157 (35.7)
Mitral	243 (55.4)
Mitral/aortic	39 (8.9)
Aortic prosthesis size (n = 196)	
21 mm	36 (18.4)
23 mm	83 (42.3)
25 mm	55 (28.0)
27 mm	16 (8.2)
29 mm	6 (3.1)
Mitral prosthesis size (n = 277)	
25 mm	31 (11.2)
27 mm	85 (30.7)
29 mm	112 (40.4)
31 mm	43 (15.5)
33 mm	6 (2.2)
Associated surgery (n = 439)	
Yes	104 (23.7)
Type of associated surgery (n = 104)	
Tricuspid plasty	61 (58.6)
Myocardial revascularization	25 (24.0)
Mitral valve plasty	5 (4.8)
Aortic valve plasty	4 (3.9)
Tricuspid valve replacement	4 (3.9)
Interventricular communication	3 (2.9)
Interatrial communication	2 (1.9)
Postoperative complications (n = 439)	
Valve thrombosis	5 (1.1)
Thromboembolism	2 (0.5)
Bleeding	4 (0.9)
Paravalvular leaks	4 (0.9)
Severe paravalvular leaks	1 (0.2)
Endocarditis	18 (4.1)
Non-structural dysfunction	6 (1.4)
Calcification	76 (17.3)
Rupture	14 (3.2)
Serious adverse events (n = 439)	
Yes	176 (40.0)
Need of reoperation (n = 439)	
Yes	49 (11.1)

### Safety Endpoints

Five complications in five patients were recorded up to 30 days after surgery: endocarditis (n = 2), bleeding (n = 2), and valve thrombosis (n = 1). Regarding SAEs during the same period, 36 events were registered: 30 deaths, two strokes, and four reinterventions. Therefore, the successful valve replacement rate was 90.7%.

In the long term, the mitral group presented the higher rates of complication and SAEs, proportionally. Calcification was the most reported complication (n = 76) while reintervention was the most reported SAE (n = 85).

Safety results are presented in Tables 3 and 4.

**Table 3:** Summary of complications and serious adverse events by replaced valve.

	Mitral		Aortic		Mitral/Aortic	
	30 days	Long term	30 days	Long term	30 days	Long term
	(n = 243)	(n = 229)	(n = 157)	(n = 146)	(n = 39)	(n = 34)
Complications						
Valve thrombosis	-	3 (1.3%)	1 (0.6%)	1 (0.7%)	-	-
Thromboembolism	-	2 (0.8%)				
Bleeding	1 (0.4%)	1 (0.4%)	-	1 (0.7%)	1 (2.5%)	-
Paravalvular leaks	-	1 (0.4%)	-	3 (2.0%)	-	-
Severe paravalvular leaks	-	1 (0.4%)	-	-	-	-
Endocarditis	1 (0.4%)	6 (2.6%)	1 (0.6%)	7 (4.8%)	-	3 (8.8%)
Calcification		53 (23.1%)	-	11 (7.5%)	-	12 (35.3%)
Rupture	-	10 (4.4%)	-	3 (2.0%)	-	1 (2.9%)
Non-structural dysfunction		3 (1.3%)		3 (2.0%)		-
Serious adverse events						
Death	14 (5.8%)	25 (10.9%)	11 (7.0%)	20 (13.7%)	5 (12.8%)	5 (14.8%)
Stroke	2 (0.81%)	6 (2.6%)	-	2 (1.4%)	-	1 (2.9%)
Reintervention	3 (1.2%)	48 (20.9%)	1 (0.6%)	25 (17.1%)	-	8 (23.5%)

**Table 4:** Successful valve replacement (freedom from complications and SAEs up to 30 days after surgery).

Valve	n (%)
Mitral	222 (91.3)
Aortic	143 (91.0)
Mitral/aortic	33 (84.6)
Total	398 (90.7)
SAEs=serious adverse events	

**Fig. 1:**
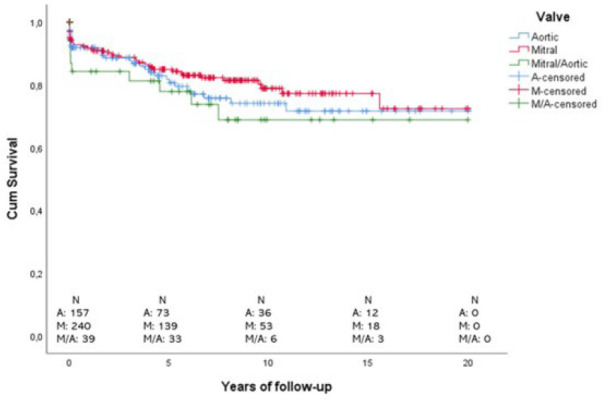
Kaplan-Meier curves of cumulative survival stratified by replaced valve. A=aortic; M=mitral; M/A=mitral/aortic.

Cumulative survival curves are presented in Figure 1. For patients submitted to aortic valve replacement, cumulative survival was 91.8% in one year, 80.6% in five years, 74.1% in 10 years, and 71.6% in 20 years. Regarding patients submitted to mitral valve replacement, it was 91.8% in one year, 84.9% in five years, 78.8% in 10 years, and 72.3% in 20 years. For patients with aortic/mitral replaced valves, cumulative survival was 84.2% in one year, 77.8% in five years, and 68.8% in both 10 years and 20 years.

**Fig. 2:**
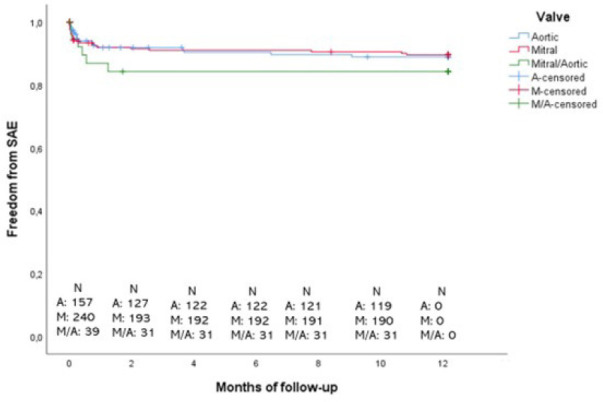
Kaplan-Meier curves of cumulative freedom from serious adverse events (SAEs) during the first year stratified by replaced valve. A=aortic; M=mitral; M/A=mitral/aortic.

Freedom from SAEs during the first year curves are presented in Figure 2. Between patients with aortic valve replacement, 88.9% were free from SAEs; patients with mitral and mitral/aortic replaced valves were 89.5% and 84.2%, respectively.

**Fig. 3:**
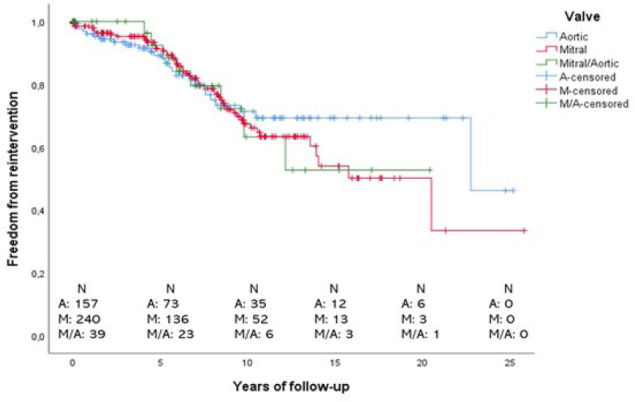
Kaplan-Meier curves of cumulative freedom from reintervention during the follow-up period. A=aortic; M=mitral; M/A=mitral/aortic.

Freedom from reintervention rate in 10 years for aortic valve patients was 71.4% for aortic valve patients, 67.4% for mitral, and 63.3% for mitral/aortic valve replacement (Figure 3).

### Efficacy Endpoint

**Fig. 4:**
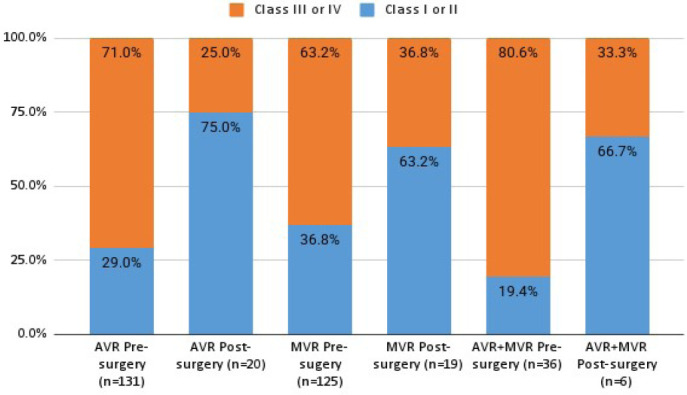
Preand post-surgery New York Heart Association classification distribution of patients submitted to aortic valve replacement (AVR), mitral valve replacement (MVR), and mitral/aortic valve replacement (AVR+MVR).

Pre-surgery NYHA classification was available for 131 patients submitted to aortic valve replacement: 29.0% were classified as class I or II, and 71.0% as class III or IV. Post-surgery rates were available for 20 patients of the same group, 75.0% being classified as class I or II, and 25.0% as class III or IV. Regarding patients of mitral valve replacement, NYHA classification at pre-surgery evaluation was available in 125 cases, being 36.8% classified as class I or II, and 63.2% as class III or IV. Post-surgery classification was available for 19 patients, and 36.8% were NYHA class III or IV, while 63.2% were classified as class I or II. In the aortic/mitral group, data was available for 36 patients regarding pre-surgery classification. Among them, 19.4% were classified as NYHA class I or II, while 80.6% were class III or IV. Post-surgery data was available for six patients, of which 66.7% were class I or II, and 33.3% were class III or IV. The incidence of preand post-surgery NYHA classification for all patients is presented in Figure 4.

## DISCUSSION

The present retrospective study reports the outcomes of 382 patients submitted to aortic, mitral, or aortic and mitral valve replacement and followed up to 26.13 years. To the authors’ knowledge, this is the study with the longest follow-up period of bovine mitral and aortic prostheses in Brazil.

The characteristics of the subjects evaluated in this study reflect the epidemiological profile of valve diseases in Brazil, where the majority of patients presents a rheumatic etiology and predominance of mitral valve impairment^[10]^. Rheumatic fever is the leading cause of heart disease in the country, contributing significantly to morbidity and mortality. Some authors have reported high rates of rheumatic etiology in Brazilian patients in need of valve replacement, specially between the years of 1970 and 1990^[11]^. Rheumatic fever has been identified as the predominant cause of valvular heart disease in young patients, with prevalence rates reaching up to 76.5%.^[11,12]^. Furthermore, rheumatic patients tend to be younger, and the degree of valve damage can lead to complications manifesting at early ages^[13]^. As observed in the present study, the young age associated with rheumatic disease leads to a high number of reoperations, with reported rates of approximately 20%^[1]^. Despite that, it is noteworthy that 70.4% of the patients did not present any postoperative complications.

Guidelines from the European Society of Cardiology (or ESC) and the American Heart Association/American College of Cardiology (or AHA/ACC) used to recommend the use of bioprostheses in patients older than 65 years of age due to their susceptibility to structural valve deterioration^[14]^. However, another characteristic of valve replacement surgeries in the Brazilian public health system is the predominance of bioprosthesis use even in young patients, comprising 50% to 84% of the surgeries. This trend is largely attributed to the socioeconomic conditions of the patients, which complicate the effective management of anticoagulation required for mechanical valves^[15]^. Consequently, this situation also results in a higher percentage of reoperations. On the other hand, long-term follow-up outcomes for patients receiving bioprosthetic implants have shown that long-term survival is not significantly influenced by the type of prosthesis but rather by other factors such as patients’ pre-existing comorbidities and risk profiles^[16]^.

The early-term results showed an all-cause 30-day mortality rate of 5.8%, 7.0%, and 12.8% for mitral, aortic, and mitral/aortic valve replacement, respectively. Those results are very similar from what is presented in the literature regarding the safety of bioprostheses. A study conducted in Brazil that evaluated 192 biological prostheses in mitral position of young patients showed a hospital mortality rate of 9.5%^[12]^. A German study, in which 1,241 aortic bovine prostheses were evaluated, reported a rate of 6.0%^[17]^. For combined surgery of both mitral and aortic valve replacement, increased morbidity and mortality have been associated with a 30-day mortality rate up to 15.5%^[18]^.

Five early-term complications (up to 30 days) were reported. Endocarditis was reported twice. It has been shown that the risk of prosthetic valve endocarditis is higher up to six weeks after surgery, being rheumatic fever an important risk factor for its development^[19]^. Bleeding was also reported in two patients, resulting in a rate of 0.4%. A study that evaluated bleeding complications related to open-heart surgery showed an incidence of in-hospital bleeding events after valve replacement surgery of 5.9%^[20]^. Valve thrombosis was another complication reported in the early term. The event happened on the same day in which the patient´s prosthetic aortic valve was replaced. Some authors have demonstrated that the risk of thromboembolic episodes is extremely higher up to 10 days after surgery in patients without anticoagulation and it can be associated with homeostatic activation, flow conditions, and patient-related risk factors^[21,22]^.

It is believed that bioprostheses have limited durability. However, the present study demonstrated that the long-term outcomes of the bovine pericardium prosthesis were acceptable. Structural complications are expected for bovine pericardium prosthesis, such as calcification^[23]^, which was the most reported complication in this study. Regarding freedom from reintervention rate, it was found to be 71.4% for aortic valve patients, 67.4% for mitral, and 63.3% for aortic/mitral valve replacement patients in 10 years. A Brazilian study of bovine pericardium prostheses in patients with a mean age of 40 years reported that freedom from reoperation rate in 11 years was 60%^[11]^. Additionally, another study conducted in Brazil between the years of 1977 and 1987 has shown a high rate of young patients in need of reintervention, reaching 75% in 14 years^[12]^.

Efficacy was aimed at being accessed through patients’ NYHA classification before and after surgery. Although it seemed to have an improvement in all three groups, post-surgery data was not available for most patients.

### Limitations

The limitations of the present study are inherent in its retrospective design, such as incomplete information on medical records. It is also important to notice that the reviewed medical records were mostly of patients submitted to valve replacement surgeries in the 1980s, and surgical technique and guidelines have evolved since then. Another known limitation is that hemodynamic data was not available for most patients, and therefore, it was not included in this study.

## CONCLUSION

The present study provides an overview of valve disease in the Brazilian public health system and the long-term performance of Braile BVP. Despite the patients’ young age and the predominance of a rheumatic etiology, safety outcomes were in accordance with those reported in the literature for bioprostheses, with acceptable complication, SAEs, and freedom from reintervention rates indicating that bioprostheses offer good prognosis. The efficacy outcome was not evaluated due to missing information on postoperative NYHA classification; however, it seemed to have an improvement on patients’ condition.
